# Theoretical framework and methodological development of common subjective health outcome measures in osteoarthritis: a critical review

**DOI:** 10.1186/1477-7525-5-14

**Published:** 2007-03-07

**Authors:** Beth Pollard, Marie Johnston, Diane Dixon

**Affiliations:** 1 School of Psychology, University of Aberdeen, Aberdeen, AB24 2UB, UK; 2 Department of Psychology, University of Stirling, Stirling, FK9 4LA, UK

## Abstract

Subjective measures involving clinician ratings or patient self-assessments have become recognised as an important tool for the assessment of health outcome. The value of a health outcome measure is usually assessed by a psychometric evaluation of its reliability, validity and responsiveness. However, psychometric testing involves an accumulation of evidence and has recognised limitations. It has been suggested that an evaluation of how well a measure has been developed would be a useful additional criteria in assessing the value of a measure. This paper explored the theoretical background and methodological development of subjective health status measures commonly used in osteoarthritis research. Fourteen subjective health outcome measures commonly used in osteoarthritis research were examined. Each measure was explored on the basis of their i) theoretical framework (was there a definition of what was being assessed and was it part of a theoretical model?) and ii) methodological development (what was the scaling strategy, how were the items generated and reduced, what was the response format and what was the scoring method?). Only the AIMS, SF-36 and WHOQOL defined what they were assessing (i.e. the construct of interest) and no measure assessed was part of a theoretical model. None of the clinician report measures appeared to have implemented a scaling procedure or described the rationale for the items selected or scoring system. Of the patient self-report measures, the AIMS, MPQ, OXFORD, SF-36, WHOQOL and WOMAC appeared to follow a standard psychometric scaling method. The DRP and EuroQol used alternative scaling methods. The review highlighted the general lack of theoretical framework for both clinician report and patient self-report measures. This review also drew attention to the wide variation in the methodological development of commonly used measures in OA. While, in general the patient self-report measures had good methodological development, the clinician report measures appeared less well developed. It would be of value if new measures defined the construct of interest and, that the construct, be part of theoretical model. By ensuring measures are both theoretically and empirically valid then improvements in subjective health outcome measures should be possible.

## Review

There has been a huge increase in the use and development of subjective health outcome measures [1]. Consequently, it is increasingly important to ensure that the measures are assessing what they intend to measure, as accurately as possible. If measures do not adequately sample the specified outcomes, or they are not accurate, then any conclusions drawn about the effectiveness of, for example, a new treatment may be misleading.

The standard approach to assessing the 'value' of a health outcome measure is to be satisfied that a measure has adequate psychometric properties in terms of reliability, validity, and responsiveness [2]. However, there are many known limitations with the most commonly reported methods of psychometric testing. For example, Cronbach's alpha [3] is widely used to evaluate internal reliability, but there is often an over-emphasis on achieving a high alpha. Selecting items for a measure based on alpha may result in almost identical items or might exclude important items, and only tap a narrow part of the underlying construct. In addition, alpha can be increased by simply increasing the number of items [2,4]. Further, the validity of a measure is often explored by correlating it with a similar existing measure. There is concern about whether the 'similar' measures are actually similar or not. A facet of this problem is known as the 'jingle-jangle fallacies': the jingle fallacy being that just because things are called the same name it does not mean that they are the same thing; the jangle refers to the issue that because things are called different things it does not necessarily mean they are different [5]. These problems are illustrated in a systematic review that found that only 16% of the identified impairment measures for rheumatic disorders were validated against a similar construct [6]. Another common problem is that claims of validity are made if a significant correlation coefficient is achieved without any reference to acceptable levels [7,8]. Finally, reliability and validity can never be proved. A single study can only provide support towards establishing reliability or validity as there needs to be an accumulation of ongoing and evolving evidence [9].

Due to the limitations of psychometric testing, other considerations may add to the assessment of the 'value' of a measure. The Scientific Advisory Committee of the Medical Outcomes Trust, 2002 [10] have suggested that the rationale for, and description of, the conceptual and measurement model of health status measures should be reported. Such theoretical and methodological criteria have generally been overlooked when evaluating health outcome measures. It is suggested therefore these criteria could be the starting point for evaluating measures *before *time, and probably costs, are involved in psychometrically evaluating the measure. Thus, an evaluation of how well a measure has been developed would appear to be a useful additional criteria in assessing the 'value' of a measure. Therefore this review explores how well measures have been developed in terms of i) theoretical framework and ii) methodological development.

### i) The theoretical framework

It is advantageous if a measure *defines *what it is supposed to be assessing (i.e. the construct of interest). For example, if we consider a measure that states it is measuring disability as a health outcome, there are many different interpretations of a what 'disability' encompasses. Disability may mean to some, limitations in physical function, but to others, it may represent a broader measure encompassing the social impact of a condition. Hence, a definition of the intended focus of a measure enhances compatibility, comparisons and understanding between studies.

Measures that are developed within a *theoretical framework *or model have the advantage of allowing underlying processes to be investigated, and interventions appropriately targeted. The dominant theoretical models of health outcomes or the consequence of disease have been the biomedical models developed by the World Health Organisation [11,12]. The most recent version is the International Classification of Functioning, Disability, and Health that identifies three distinct outcomes, impairment, activity limitations and participation restrictions [12]. Using this model, we may find that analgesics influence all three outcomes, whereas modifying the structure of the home might only alter activity limitations. Failure to adequately measure each distinguishable outcome might result in failure to detect benefit or harm occurring due to an intervention or to a disease. Further, with distinguishable outcomes, it is possible to postulate relationships between them, e.g. in the analgesic example, pain relief might affect impairment with consequent reductions in activity limitations. In this review, considerations are given to whether the underlying construct has been defined and whether the construct is part of a theoretical model.

### ii) The methodological development

The use of a standard *scaling *procedure (i.e. the method of attributing numerical values to responses) is advantageous as it prescribes a standard, theoretically sound method for developing and scoring measures. Standard scaling methods usually start by collecting a large number of items, and then use defined methods to reduce the number of items, attach a response format, and score the final scale. The most common standard scaling techniques in health status measures have been derived from the scaling of attitudes – Likert [13], Guttman [14] and Thurstone scaling [15]. These methods ensure that the scoring, scaling, and the response format for items will be consistent. For example, if a Likert scaling technique is used then all items will conform to a Likert scale with Likert response formats (5 point with agree to disagree response stems) and use an additive scoring method, whereas Guttman scaling requires a binary response format, and the score reflects the 'highest' item endorsed. However, if only some aspects of the scaling method are followed, it is possible that problems with the scale will arise. For example, it has been shown that problems with a 'gold standard' measure, the Sickness Impact Profile, were due to an inconsistency between the scoring method (additive) and the scaling method (Thurstone scaling) [16]; as a result, an individual with small limitations could have a higher score than someone who was completely incapacitated.

If a standard method is not implemented, it is preferable if the method for *selecting items *is broad enough to sample the full range and not restricted to just one source or domain. For example, in a thorough selection process items, may have been derived from previous measures, research literature, expert judges, patients, and healthy individuals. The resultant pool may then be reduced by going through a systematic sorting or *item reduction *process. The resultant items may then be explored empirically through item analysis, enabling poor items to be identified and eliminated from the final measure.

Therefore in this review, considerations are given to the scaling strategy, item generation and reduction, scaling, response format, and scoring method of each of the measures. Additionally, the explanations given for the rationale for the response categories and scoring method are reviewed.

In summary, the aim of this review is to explore the theoretical framework and methodological development of common subjective health outcome measures using the criteria specified in Table 1. The context of osteoarthritis has been chosen as the focus of this review.

**Table 1:** Criteria used to assess the theoretical framework and methodological development of health outcome measures

**Theoretical framework**
1. What construct is being measured?
2. Has the construct been defined?
3. Was the construct part of a (specified) theoretical model?
**Methodological development**

4. What scaling strategy was adopted?
5. How were the items generated (to tap the construct)?
6. How was item reduction conducted?
7. What was the response format?
8. What was the scoring method?

## Methods

### Measures

The measures selected were commonly used to assess subjective health outcome in hip or knee osteoarthritis (OA). The measures were identified as part of a review of interventions used for the treatment of OA [17]. In addition, citation-based searches (using Web of Science) for other subjective health outcome measures were undertaken to identify any very widely used measures not already selected. Nine hundred and forty abstracts were examined and all named measures noted. Any measure with 10 or more citations was included in this review.

This resulted in the addition of two measures: the Hospital for Special Surgery knee score (HSS) [18] and the Merle d'Aubigne Hip Rating [19]. An in depth theoretical review of one of the measures, the Sickness Impact Profile, [20] has already been carried out [16], and so was not included here. This resulted in 10 disease-specific measures (clinician report or patient self-report) and 4 generic measures (all patient self-report). The measures are specified in Table 2.

**Table 2:** Outcome instruments assessed in this study

**Generic**
Patient self-report: EuroQol [21]
Medical Outcomes Study Short Form-36 (SF-36) [22-25]
McGill Pain Questionnaire (MPQ) [26-28]
World Health Organisation Quality of life Assessment (WHOQOL) [29,30]
**Disease Specific – Clinician report**

American Knee Society Score (AKS) [31]
Harris Hip Score [32]
Hospital for Special Surgery Knee Score (HSS) [18]
Lequesne Hip and Knee Indices [33]
Merle d'Aubigne Hip Rating [19]
**Disease Specific – Patient self-report**

Arthritis Impact Measurement Scale (AIMS) [34,35]
Disease Repercussion Profile (DRP) [36-38]
Health Assessment Questionnaire- Disability Index (HAQ-DI) [39-42]
Oxford Hip and Knee Questionnaires [43,44]
Western Ontario and MacMaster Universities Osteoarthritis Index (WOMAC) [45-48]

### Analysis

A literature search was conducted for published papers relating to the development of each measure and they were examined (a complete search may not have been carried out where papers were published prior to electronic database searches limits, where papers were unavailable in English, or where the paper could not be traced). The focus of this review was on the original measure rather than modified versions (e.g. short forms).

The information extracted from the literature for this review was:

a) For the basic description of measures: the number of items and item content areas.

b) For the theoretical framework: was the underlying construct defined and was the construct part of a theoretical model?

c) For the methodological development: what was the scaling strategy, how were the items generated and reduced, what was the response format and what was the scoring method?

## Results

A summary of the basic measure information is in 'Additional file 1' and a summary of the review is in 'Additional file 2'.

### i) Theoretical framework

The clinician report measures stated what the measure was about but none defined what it was supposed to be assessing. These measures also lacked an underlying theoretical framework. The American Knee Society Score (derived to measure knee and patient function), Harris Hip Score (pain and functional capacity), Hospital for Special Surgery Knee Score (disability), Lequesne Hip and Knee Indices (an indices of severity of disease), Merle d'Aubigne Hip Rating (function of the hip) are all measures which, while of value clinically, did not have a well defined construct, nor were they derived from a strong theoretical framework.

Some self-report measures were based on conceptual frameworks proposed by the author(s) of the measure. The McGill Pain Questionnaire (MPQ) was based on a Melzack's theory of pain [49]. This review focuses on the Pain Rating Index (PRI) and the present pain intensity (PPI) item of the McGill Pain Questionnaire. The Health Assessment Questionnaire (HAQ) was based on a hierarchical model of death, disability, discomfort, drug toxicity and dollar cost [39]. This most commonly used part of the HAQ, the Disability Index (HAQ-DI) is focussed on in this review. Much consideration was given to the conceptual meaning of handicap in the process of developing the Disease Repercussion Profile. The Disease Repercussion Profile measures individualised patient-perceived handicap in a broader manner than the WHO defined dimensions of handicap [11]. Other measures were based on an existing defined construct. The SF-36 was derived to measure health status based on the identification and definition of five generic health concepts [22] plus two other concepts identified from empirical evidence [23]. The Arthritis Impact Measurement Scale was developed to reflect the WHO definition of health [50], and the WHOQOL from the definition of quality of life devised by the WHOQOL group [30].

Other measures stated the construct measured but without explicit definition. The EuroQol was developed as a standardised non-disease specific measure for describing and valuing health-related quality of life [21]. The dimensions were selected primarily from existing health status measures. The WOMAC was based on the objective of defining the dimensionality of pain and disability, with five dimensions being initially identified [45]. The final version had three subscales of pain, stiffness, and physical function [46]. The underlying aim of the Oxford Hip and Knee Questionnaires was to measure "patients' perception of a single disease entity" [43].

Thus although three measures defined the construct of interest, *no *measure was based on both a defined construct and a theoretical framework.

### ii) Methodological development

#### Scaling strategy

Six of the fourteen measures appeared to use standard psychometric scaling methods. The stated scaling methodology of the SF-36, WOMAC and WHOQOL was Likert scaling. The WOMAC could, alternatively, be implemented using a 0–100 mm visual analogue scale for each item, with descriptive anchors of none and extreme. A numeric rating scale version of the WOMAC has also been developed, with response categories between 0 (none) and 10 (extreme) [48]. While the authors of the Oxford Hip and Knee Questionnaires did not state that Likert scaling was used, the resultant questionnaire had the appearance of a Likert-type scale. Two scaling methods were used for the Arthritis Impact Measurement Scale: first, items were grouped into subscales and each subscale was examined using Guttman scaling procedures, and then Likert scaling was used to form an additive scale for each subscale. Thurstone's Categorical Judgement model [51] was used to obtain weightings of pain intensity for each descriptor of pain in the McGill Pain Questionnaire-PRI. This procedure results in an interval scale. The McGill Pain Questionnaire-PPI was a single item with five response categories that were considered equally far apart as to represent an interval scale.

An econometric scaling method was used for the development of the EuroQol. This method involved subjects rating health states (from combining different levels from each item) and results in values being attached to each health state. The Disease Repercussion Profile used a combination of open questions and 10-point graphical rating scales to create a graphical profile score. The HAQ-DI did not appear to have been developed using a standard scaling technique.

None of the clinician report measures appeared to have been developed using a standard scaling technique nor did they explain their scaling strategy.

#### Item generation technique

A range of techniques was used to generate the items within a measure. There was no information on the item selection techniques for the Harris Hip Score, Hospital for Special Surgery Knee Score, Lequesne Hip and Knee Indices and Merle d'Aubigne Hip Rating. The items for the American Knee Society Score were generated by consensus by members of the American Knee Society. Some measures were based on items from existing instruments (Arthritis Impact Measurement Scale, EuroQol, HAQ-DI, SF-36). Some items were selected from literature, e.g. McGill Pain Questionnaire. Others started by gathering items from patients, e.g. Oxford Hip and Knee Questionnaires, WOMAC and Disease Repercussion Profile. Some measures took a comprehensive approach and used all these techniques and additional ones (e.g. extensive focus groups and question writing panels were additionally used for the WHOQOL). In summary, the method of item generation for the patient self-report measures was generally comprehensive, with most measures using appropriate methods to generate a pool of items that cover the domain of interest. In contrast, there was little information about the choice of items in the clinician report measures.

#### Item reduction

The Arthritis Impact Measurement Scale, McGill Pain Questionnaire, WHOQOL and WOMAC used psychometric methods of item reduction to reduce the number of items. The SF-36 used specific methods to construct short-form measures from the 'parent' longer Medical Outcomes Study measure [23,52]. The method details were not found; however, if the methods were similar to those for the SF-20 [52] then it would imply comprehensive testing where item-scale correlations, reliability and validity were examined. Subsequently, the Likert scaling assumptions of the SF-36, were explored with all scales passing tests for item-internal consistency, item-discrimination, and internal consistency of each scale score [24]. The main item reduction for the HAQ-DI was carried out by correlational analyses that identified redundant items [40]. The methods of item reduction for the Oxford Hip and Knee Questionnaires and EuroQol were not explained in detail in the published literature. The item reduction procedures were described in detail for the measures where a stated psychometric scaling strategy was followed, illustrating the advantage of using a psychometric scaling method with an explicit predefined methodology.

#### Response formats

The Disease Repercussion Profile used open questions for each domain, with severity being rated on a ten point graphical rating scale. For the McGill Pain Questionnaire-PRI, the respondents select from each of the 20 categories, the individual descriptive words that best represent their pain. If none of the words in a category apply then the respondent leaves the category out. For the present pain intensity item, the respondent selects one of five response categories.

All the other twelve measures had ordered response categories with the Arthritis Impact Measurement Scale & the EuroQol additionally including a visual analogue scale. Six of these twelve measures had items with different numbers of response categories (American Knee Society Score, Lequesne Hip and Knee Indices, Hospital for Special Surgery Knee Score, Harris Hip Score, SF-36 & the Arthritis Impact Measurement Scale with between 1 and 6 response categories depending on the measure and item). However, the number of response categories was only discussed for the SF-36 and then only for some items [23]. The other six measures had the same number of response categories for all the items throughout the measure (EuroQol, HAQ-DI, Merle D'Aubigne Hip Rating, Oxford Hip and Knee Questionnaires, WHOQOL, WOMAC). Of these, only the WOMAC and HAQ-DI had the same response continuum (i.e. same wording) for all the items. The HAQ-DI response formats were based on the American Rheumatism Association (ARA) functional classes.

Therefore most of the measures used ordinal (ordered) response formats but there was little consistency of the response format and response continuum within measures. There is much discussion on the problems in performing arithmetic operations and statistical analysis on ordinal scales, mainly due to the unknown interval between categories [53,54]. The PRI index of the McGill Pain Questionnaire was the only measure on an interval scale and therefore was without these problems. Likert scales are ordinal, although there is much debate as to whether they can be assumed to be interval (i.e., with equal intervals between responses [2]). The response format for the Likert-type measures (SF-36, WOMAC, WHOQOL, Arthritis Impact Measurement Scale, Oxford Hip and Knee Questionnaires) were not true Likert scales as the response continuum was not 'agree' to 'disagree'. This may have an impact on the resultant scale as any changes in the response categories, e.g., changing the usual agree-disagree to favourable-unfavourable, may have an impact on the intervals between the categories. In addition, all the items within a true Likert scale usually have either five or seven response categories, but the Arthritis Impact Measurement Scale and the SF-36 did not use a constant number of response categories, which again may impact the scale. However, it is not clear whether these changes from a traditional Likert scale have a significant impact as there was empirical support for the scaling assumptions of traditional Likert scales in the SF-36 subscales [24].

#### Scoring method

The McGill Pain Questionnaire-PRI used three possible scoring methods for the list of pain descriptors: the number of items chosen (NWC), the mean scale values (PRI(S)), or the summed rank values of items chosen ((PRI(R)). An alternative weighted-rank method of scoring was also developed [28]. The PPI score was simply the value selected from the 1–5 response scale. The Disease Repercussion Profile used profile scores, where the handicap rating for each domain was plotted on a bar chart to obtain a handicap profile for each patient.

Two measures containing items with different numbers response categories addressed this in their scoring. The Arthritis Impact Measurement Scale used a standardised additive scale. The SF-36 recalibrated the additive scores for linearity and transformed the scores. The American Knee Society Score, Harris Hip Score, Hospital for Special Surgery Knee Score, and Lequesne Hip and Knee Indices (all with varying numbers of response categories) used summated scale systems with the Hospital for Special Surgery Knee Score and American Knee Society Score having items that result in deductions from the point score, e.g., Hospital for Special Surgery Knee Score uses a one point deduction for using a cane. It is unclear how this scoring method was derived and why responses to certain items were allocated their particular points with some items having more weighting than others.

The scoring of the measures with constant numbers of response categories varies; an additive score was used for the Likert-type scales of the Oxford Hip and Knee Questionnaires and WHOQOL. An additive scale is also most commonly used for the WOMAC, however other weighting and aggregation methods were proposed (i.e. normalisation, pooled index, weighting by relative importance, response criteria) [48]. In addition, the WOMAC can be scored using a signal method where patients are asked to select the most important item from each subscale. However, there are concerns about the stability of using the signal method and is not currently recommended [47]. The score for the HAQ-DI items was based on the highest score on any item within each of the eight subscales. The subscale scores were adjusted to take account of the use of aids. An overall disability score was calculated as the average of the subscale scores. The EuroQol could be scored as a profile or a weighted health index based on a table of values from general population samples. A table was used for the Merle D'Aubigne Hip Rating to allow classification of the functional grading of the hip, and an algorithm was provided to calculate improvement after surgery on the hip.

Three of the measures (Oxford Hip and Knee Questionnaires, Merle D'Aubigne Hip Rating and Lequesne Hip and Knee Indices) had only an overall score. All the others also had subscale scores. The SF-36 and American Knee Society Score only had subscale scores and not an overall score. All other measures had an overall score.

In sum, the measures use a wide range of scoring procedures, from the complex weightings in the EuroQol to the simple method of the HAQ-DI (using the highest score within each subclass) that does not fully utilise all the information collected. Jenkinson, 1991 [55] demonstrated that complex weighting methods gain little over a simple scoring system, and thus a simple additive method is generally recommended

## Discussion

Although most measures gave some indication of what they were measuring, few defined the construct or linked it to a theoretical model. The clinician report measures were generally the poorest measures in this respect. The Arthritis Impact Measurement Scale, SF-36 and WHOQOL defined their construct of interest, but it was not related to a theoretical model. The Disease Repercussion Profile and McGill Pain Questionnaire discussed, in detail, their underlying construct (although without a stated definition of terms).

The measures that appeared to have the weakest methodological development were the clinician report measures with none defining a scaling strategy. The item selection for the American Knee Society Score was by 'consensus' with no other clinician report measure describing the item selection method. No clinician report measure explained their choice of response categories or scoring method.

Of the patient self-report measures, only the McGill Pain Questionnaire-PRI was completely developed from a standard scaling procedure. The McGill Pain Questionnaire-PRI was also the only measure with an interval scale, and hence has mathematical and statistical advantages over all the other measures. The other measures that appeared to use a standard scaling procedure were the Arthritis Impact Measurement Scale, Oxford Hip and Knee Questionnaires, SF-36, WOMAC and WHOQOL. The Disease Repercussion Profile and EuroQol used alternative scaling methods, while the HAQ-DI did not appear to have a specific scaling strategy.

The method of item selection was generally good for the patient self-report measures, although the item reduction methods were not always explained, except for those that used a defined scaling procedure. In addition, the reasoning for the choice of response formats was not often explained. The scoring method was generally appropriate for the scaling method (where used) and for the item response format, although the HAQ-DI used a method that did not maximise the information available.

In summary, the clinician report measures were poor in terms of both their theoretical framework and methodological development. The patient self-report measures appeared to have acceptable methodological development, although there were some limitations with the HAQ-DI. However only the Arthritis Impact Measurement Scale, SF-36 & WHOQOL defined the construct that they were assessing and no measure was part of a theoretical model.

While this review has focussed on specific theoretical criteria, it is appreciated that there are other theoretical factors that should be explored such as the rationale for the grouping of items into subscales.

This review was based on peer reviewed published literature on the development of the measures, and some theoretical aspects of the development may have been unpublished. However, it is important for users of measures to have this background information, and electronic publishing methods may facilitate access to this more detailed information.

The review was based on OA measures that were frequently referenced in the literature and hence some of the newer measures such as the Knee injury and Osteoarthritis Outcome Score (KOOS) [56], Hip disability and Osteoarthritis Outcome Score (HOOS) [57], Musculoskeletal Functional Assessment Questionnaire (MFA) [58] were not evaluated here. The uptake and utility of these newer measures remains to be seen.

Further, this review has focussed on measures used as outcome for osteoarthritis and different conclusions may be reached for other health outcomes or for other conditions. Where outcomes are psychologically theorised, e.g. mood measurements such as anxiety, it is likely that they are more theoretically based and would have used development procedures derived from psychometric theory. However, many health outcomes, especially those involving self-report, require a similar level of attention to measurement issues. They assess patients' experience of their health condition and healthcare and therefore relate to unobservable phenomena rather than phenomena that can be observed by others. One reason for the limited development of some of the measures in osteoarthritis may be that such outcomes have not been articulated as psychological in nature and as a result not subjected to normal psychometric evaluation.

## Conclusion

This review has highlighted the general lack of attention given to the theoretical framework of the health outcome measures. It would be valuable if new measures could define what they are measuring and be a construct within a theoretical model.

The review also demonstrates the large variation in the methodological development of commonly used measures in OA. While patient self-report measures had, in general, good methodological development, this review has also highlighted the relatively poor development of clinician report measures.

It is suggested that to improve the quality and performance of new measures, the foundations of their theoretical development should be considered before psychometric evaluation is performed. By ensuring measures are both theoretically and empirically valid, improvements in subjective health outcome measures should be possible.

## Competing interests

The author(s) declare that they have no competing interests.

## Authors' contributions

BP participated in the conception and design of the study, the analysis and the drafting and revision of the manuscript. MJ participated in the conception and design of the study and the drafting and revision of the manuscript. DD contributed to the interpretation of the data and revision of the manuscript. All authors read and approved the final manuscript.

## Supplementary Material

Additional file 1 Summary information on each measure. Table of summary information on each measure (number of items and item content areas) Click here for file

Additional file 2 Summary of the theoretical review. Summary table of the review Click here for file

## References

[B1] Jenkinson C, Bardsley M, Lawence K, Jenkinson C (1994). Measurement in subjective health assessment: themes and prospects. Measuring Health and Medical Outcomes.

[B2] Streiner D, Norman GR (1989). Health measurement scales: a practical guide to their development and use.

[B3] Cronbach LJ (1951). Coefficient alpha and the internal structure of tests. Psychometrika.

[B4] Fitzpatrick R, Davey C, Buxton MJ, Jones DR (1998). Evaluating patient-based outcome measures for use in clinical trials. Health Technol Assess.

[B5] Pedhazur EJ, Schmelkin LP (1991). Measurement, Design and Analysis.

[B6] Swinkels RAHM, Bouter LM, Oostendorp RAB, Swinkels-Meewisse IJCM, Dijkstra PU, de Vet HCW (2006). Construct validity of instruments measuring impairments in body structures and function in rheumatic disorders: Which constructs are selected for validation? A systematic review. Clin Exp Rheumatol.

[B7] Bowling A (1991). Measuring health: a review of quality of life measurement scales.

[B8] McDowell I, Newell C (1987). Measuring health: a guide to rating scales and questionnaires.

[B9] Messick S (1995). Validity of psychological assessment – validation of inferences from persons responses and performances as scientific inquiry into score meaning. Am Psychol.

[B10] (2002). Assessing health status and quality-of-life instruments: Attributes and review criteria. Qual Life Res.

[B11] World Health Organisation (1980). International Classification of Impairments, Disabilities and Handicaps.

[B12] World Health Organisation (2001). The International Classification of Functioning, Disability and Health.

[B13] Likert R (1932). A technique for the measurement of attitudes. Archives of Psychology.

[B14] Guttman LL (1944). A basis for the scaling of quantitative data. Am Sociol Rev.

[B15] Thurstone L, Chave EJ (1929). The measurement of attitude.

[B16] Pollard B, Johnston M (2001). Problems with the Sickness Impact Profile: a theoretically based analysis and a proposal for a new method of implementation and scoring. Soc Sci Med.

[B17] Chard JA, Tallon D, Dieppe PA (2000). Epidemiology of research into interventions for the treatment of osteoarthritis of the knee joint. Ann Rheum Dis.

[B18] Ranawat CS, Insall J, Shine J (1976). Duo-Condylar knee arthroscopy. Clin Orthop Relat Res.

[B19] D'Aubigne RM, Postel M (1954). Hip arthroplasty and acrylic prosthesis. Journal of Bone and Joint Surgery-American Volume.

[B20] Bergner M, Bobbitt RA, Carter WB, Gilson BS (1981). The Sickness Impact Profile – development and final revision of a health-status measure. Med Care.

[B21] (1990). Euroqol-A New Facility for the Measurement of Health-Related Quality-Of-Life. Health Policy.

[B22] Ware JE (1987). Standards for Validating Health Measures – Definition and Content. J Chronic Dis.

[B23] Ware JE, Sherbourne CD (1992). The MOS 36-item short-form health survey (SF-36) .1. Conceptual-framework and item selection. Med Care.

[B24] McHorney CA, Ware JE, Lu JFR, Sherbourne CD (1994). The MOS 36-Item short-form health survey (SF-36) .3. Tests of data quality, scaling assumptions, and reliability across diverse patient groups. Med Care.

[B25] Ware JE, Snow KK, Kosinski MK, Gandek BG (1993). SF-36 Health Survey: Manual and interpretation guide.

[B26] Melzack R, Torgerson WS (1971). On the language of pain. Anesthesiology.

[B27] Melzack R (1975). The McGillI Pain Questionnaire: major properties and scoring methods. Pain.

[B28] Melzack R, Katz J, Jeans ME (1985). The Role of Compensation in Chronic Pain – Analysis Using A New Method of Scoring the Mcgill Pain Questionnaire. Pain.

[B29] (1995). The World-Health-Organization Quality-Of-Life Assessment (Whoqol) – Position Paper from the World-Health-Organization. Soc Sci Med.

[B30] (1998). The World Health Organisation quality of life assessment (WHOQOL): Development and general psychometric properties. Soc Sci Med.

[B31] Insall JN, Dorr LD, Scott RD, Scott WN (1989). Rationale of the knee-society clinical rating system. Clin Orthop Relat Res.

[B32] Harris WH (1969). Traumatic Arthritis of the hip after dislocation and acetabular fractures: treatment by Mold arthroplasty. Journal of Bone and Joint Surgery-American Volume.

[B33] Lequesne MG, Mery C, Samson M, Gerard P (1987). Indexes of Severity for Osteo-Arthritis of the Hip and Knee – Validation Value in Comparison with Other Assessment Tests. Scand J Rheumatol Suppl.

[B34] Meenan RF, Gertman PM, Mason JH (1980). Measuring health status in arthritis – The Arthritis Impact Measurement Scales. Arthritis Rheum.

[B35] Meenan RF, Gertman PM, Mason JH, Dunaif R (1982). The Arthritis Impact Measurement Scales – Further Investigations of A Health-Status Measure. Arthritis Rheum.

[B36] Carr AJ, Thompson PW (1994). Towards a measure of patient-perceived handicap in rheumatoid-arthritis. Br J Rheumatol.

[B37] Carr AJ (1996). A patient-centred approach to evaluation and treatment in rheumatoid arthritis: The development of a clinical tool to measure patient-perceived handicap. Br J Rheumatol.

[B38] Carr AJ (1999). Beyond disability: measuring the social and personal consequences of osteoarthritis. Osteoarthritis Cartilage.

[B39] Fries JF, Spitz P, Kraines RG, Holman HR (1980). Measurement of patient outcome in arthritis. Arthritis Rheum.

[B40] Fries JF, Spitz P, Young DY (1982). The dimensions of health outcomes: The Health Assessment Questionnaire, disability and pain scales. J Rheumatol.

[B41] Ramey DR, Raynauld J-P, Fries JF (1992). The Health Assessment Questionnaire 1992. Arthritis Care Res.

[B42] Bruce B, Fries JF (2003). The Stanford Health Assessment Questionnaire: A review of its history, issues, progress and documentation. J Rheumatol.

[B43] Dawson J, Fitzpatrick R, Carr A, Murray D (1996). Questionnaire on the perceptions of patients about total hip replacement. J Bone Joint Surg Br.

[B44] Dawson J, Fitzpatrick R, Murray D, Carr A (1998). Questionnaire on the perceptions of patients about total knee replacement. J Bone Joint Surg Br.

[B45] Bellamy N, Buchanan WW (1986). A preliminary evaluation of the dimensionality and clinical importance of pain and disability in osteoarthritis of the hip and knee. Clin Rheumatol.

[B46] Bellamy N, Buchanan WW, Goldsmith CH, Campbell J, Stitt LW (1988). Validation-study of WOMAC – A health status instrument for measuring clinically important patient relevant outcomes to antirheumatic drug-therapy in patients with osteo-arthritis of the hip or knee. J Rheumatol.

[B47] Barr S, Bellamy N, Buchanan WW, Chalmers A, Ford PM, Kean WF, Kraag GR, Gerecz-Simon E, Campbell J (1994). A Comparative study of signal versus aggregate methods of outcome measurement based on the WOMAC Osteoarthritis Index. J Rheumatol.

[B48] Bellamy N (2004). Womac Osteoarthritis Index User Guide VII.

[B49] Melzack R (1971). On the language of pain. Anesthesiology.

[B50] World Health Organisation (1958). The First Ten Years of the World Health Organisation.

[B51] Torgerson WS (1958). Theory and Methods of Scaling.

[B52] Stewart AL, Ware JE (1999). Measuring functioning and well being: the medical outcomes study approach.

[B53] Merbitz C, Grip JC (1989). Ordinal scales and foundations of misinference. Arch Phys Med Rehabil.

[B54] Wright BD, Linacre JM (1989). Observations Are Always Ordinal – Measurements, However, Must be Interval. Arch Phys Med Rehabil.

[B55] Jenkinson C (1991). Why are we weighting – A critical-examination of the use of item weights in a health-status measure. Soc Sci Med.

[B56] Roos EM, Roos HP, Lohmander LS, Ekdahl C, Beynnon BD (1998). Knee injury and osteoarthritis outcome score (KOOS) – Development of a self-administered outcome measure. J Orthop Sports Phys Ther.

[B57] Klassbo M, Larsson E, Mannevik E (2003). Hip disability and osteoarthritis outcome score – An extension of the Western Ontario and McMaster Universities Osteoarthritis Index. Scand J Rheumatol.

[B58] Engelberg R, Martin DP, Agel J, Obremsky W, Coronado G, Swiontkowski MF (1996). Musculoskeletal function assessment instrument: Criterion and construct validity. J Orthop Res.

