# Supplementary material for: Theoretical framework and methodological development of common subjective health outcome measures in osteoarthritis: a critical review
Source: Health Qual Life Outcomes. 2007 Mar 7;5:14. doi: 10.1186/1477-7525-5-14 (PMC1832179; doi:10.1186/1477-7525-5-14)
Supplement: Additional file 1 — Summary information on each measure. Table of summary information on each measure (number of items and item content areas) [file 1477-7525-5-14-S1.doc]

# Additional file 1: Summary information on each measure

| **Generic measures** | **No. of items** | **Item content areas** |
| --- | --- | --- |
| **Patient Self report** |  |  |
| EuroQol | 6 | Mobility, self-care, usual activities, pain/discomfort, anxiety/depression  + Visual analogue scale for patients to rate their health status |
| McGill Pain  Questionnaire (MPQ) | PRI: 78 words in 20 categories  PPI: 1 | Pain Rating Index (PRI)-What does pain feel like? - 20 groups of words.  Present Pain Intensity (PPI)-Which word describes pain right now?  (Other sections not reviewed: Location of pain (drawings), pain changes over time, intensity of other pain experiences) |
| SF-36 | 36 | Subscales: Physical functioning, Role limitations - physical, Social functioning, Bodily pain, Mental health, Role limitation – emotional, Vitality, General health  + Health transition (1 item) |
| WHOQOL | 100 | Subscales: Physical health (incorporating independence), Psychological (incorporating spirituality), Social Relationships, Environment. |
|  |  |  |
| **Disease-specific measures** |  |  |
| **Clinician report** |  |  |
| American Knee Society Score (AKS) | 9 | Knee rating subscale: Pain, Range of motion, Stability. Deductions for flexion contracture, deformity, extension lag and misalignment.  Knee function subscale: Function. Deductions for walking aids. |
| Disease Repercussion Profile (DRP) | 6 | Functional activities, social activities, socio-economic status, relationships, emotional well-being, body image. |
| Harris Hip Score | 9 | Pain, Function, Absence of deformity and Range of motion. |
| Hospital for Special Surgery Knee Score (HSS) | 7 | Pain, Function, Range of motion, Muscle strength, Flexion deformity, Instability. Deductions for external aids, residual extension lag and valgus and varus deformities. |
| Lequesne Hip and Knee Indices | 10 hip  10 knee | Pain/Discomfort, Maximum distance walked, Activities of daily living. |
| Merle d’Aubigne Hip Rating | 3 | Pain, Mobility, Ability to walk. |
|  |  |  |
| **Patient self report** |  |  |
| Arthritis Impact Measurement Scale  (AIMS) | 45 | Subscales: Mobility, Physical activity, Dexterity, Household activity, Social activity, Activities of daily living, Pain, Depression and Anxiety. |
| Health Assessment Questionnaire Disability Index (HAQ-DI) | 20 | Subscales: Activity, Arising, Dressing, Eating, Grip, Hygiene, Reach, Walking. |
| Oxford Hip and Knee Questionnaires | 12 hip  12 knee | Pain, function |
| WOMAC | 24 | Subscales: Functional activities, Pain, Stiffness |
