# Supplementary material for: Theoretical framework and methodological development of common subjective health outcome measures in osteoarthritis: a critical review
Source: Health Qual Life Outcomes. 2007 Mar 7;5:14. doi: 10.1186/1477-7525-5-14 (PMC1832179; doi:10.1186/1477-7525-5-14)
Supplement: Additional file 2 — Summary of the theoretical review. Summary table of the review [file 1477-7525-5-14-S2.doc]

**Additional file II: Summary of the theoretical review**

| **Generic  measures** | **Underlying construct** | **Scaling strategy** | **Item generation**  **Technique** | **Item reduction** | **Response formats** | **Scoring method** |
| --- | --- | --- | --- | --- | --- | --- |
| EuroQol | Health-related quality of life – not defined  Standardised non-disease specific- single index | Health state valuation | Meta analysis of existing health status questionnaires | Descriptors selected to ‘cover as many as possible of the domains frequently covered by others’ and cover wide range of severity within each domain - no further details in literature | 3 response categories  Different wordings  VAS: (0-100mm) | Profile or weighted health index |
| McGill Pain  Questionnaire  (MPQ) | Based on Melzack’s theory of pain | Pain Rating Intensity (PRI) -Equal appearing intervals for words & groupings  Present Pain Intensity (PPI) - 5 point scale | 120 words from questionnaires & literature | PRI: Non-agreement of judges & equal appearing intervals=78 words | PRI: Select one word from each group if any applicable.  PPI: 1-5 | PRI: 3 originally proposed: No. of items (NWC); Mean scale values (PRI(S));  Rank of values (PRI(R))  Subsequently: weighted rank method developed  PPI: scale value |
| SF-36 | Physical and mental concepts and multiple manifestations of well being and health =  8 health concepts with  5 defined | Likert method of summated ratings | Review of existing measures.  Items selected to reproduce the parent scale of the Medical Outcomes Survey (MOS) - 245 items. | Full MOS as criterion  & psychometric standards considered  Difficult to find detail of item reduction to 36 items.  Likert assumptions tested for the 36 items | 2-6 response categories  Different wordings | Summated scores for each dimension Recalibrated for linearity and  transformed |
| WHOQOL | WHOQOL group defined quality of life then worldwide (15 centres) generated facets by focus groups | Likert | From focus groups, question writing panels in each centre. Maximum of 12 items per facet – all items pooled (across centres). This resulted in 1800 items with 1000 dissimilar items. | Principal from each centre ranked each item on importance as judged from focus group discussions  - 236 items /29 facets in pilot  Then tested on 300 subjects in each centre - psychometric methods used to reduce to 24 facets.  4 items per facet to be able to test reliability | 5 response categories  Different wordings | Sum for each facet.  6 domain scores:  from EFA and CFA  physical, psychological, social, environment, spiritual, independence  Later reduced to 4 domains (1^st^ four above) |

| **Disease specific**  **Measure** | **Underlying construct** | **Scaling strategy** | **Item generation**  **Technique** | **Item reduction** | **Response format** | **Scoring method** |
| --- | --- | --- | --- | --- | --- | --- |
| **Clinician report** |  |  |  |  |  |  |
| American Knee Society  Score (AKS) | Not defined - Knee rating and function | No details in literature | Consensus of knee society | No details in literature | 3-7 response categories  Different wordings | Knee & Function score - additive  100 points with deductions |
| Harris Hip  Score | Not defined- function & capacity | No details in literature | No details in literature | No details in literature | 1-6 response categories  Different wordings | Additive 100 points  Separate method for Range of Motion |
| Hospital for Special Surgery  Knee Score (HSS) | Not defined -Knee disability | No details in literature | No details in literature | No details in literature | 1-9 response categories  Different wordings | Additive points with deductions |
| Lequesne Hip & Knee Indices | Not defined- severity index | No details in literature | No details in literature | No details in literature | 1-8 response categories  Different wording | Additive |
| Merle D'Aubigne Hip Rating | Not defined -Function of the hip/improvement | No details in literature | No details in literature | No details in literature | 6 response categories  Different wordings | For function: table of scores to grade hip  For improvement: based on sum of differences |
| **Self report** |  |  |  |  |  |  |
| Arthritis Impact Measurement Scale (AIMS) | WHO definition of health | Guttman for item selection then Likert for response format and scoring | Previous questionnaires  Initially 55 items | Items examined to produce optimal Guttman scales - 46 items - 1 item (sex) dropped in subsequent version | 2-6 response categories  Different wordings | Standardised summated scores:  9 dimensions and overall |
| Disease Repercussion Profile (DRP) | Individualised measure:  Individual function, social, psychological, emotional and economic disadvantage i.e. ‘patient perceived’ handicap | Graphical rating scale | ‘Grounded theory’ approach based on a survey on impact of RA on 458 patients | Responses from patients could be separated into 6 domains | Open questions  Severity rated on 10 point graphic rating scales | A handicap profile obtained by plotting the handicap rating for each domain on a bar chart. |
| Health Assessment Questionnaire  (HAQ) | Hierarchical model : death, disability, discomfort, drug toxicity and dollar cost | No details in literature | Existing instruments – range of questionnaires considered 200 items.  Disability Index: 62 items | Pilot of 62 items  Pre-tested and revised repeatedly  Redundant items eliminated. | Disability Index:  8 components 20 items (all 4 response categories - same wording: based on ARA functional classes)  Checklist for ‘use of aids/help needed’ items | Disability Index:  Average of highest score for any question within component (adjusted for use of aids)  Based on the 8 components (0-3). |

| **Disease specific**  **Measure** | **Underlying construct** | **Scaling strategy** | **Item generation**  **Technique** | **Item reduction** | **Response format** | **Scoring method** |
| --- | --- | --- | --- | --- | --- | --- |
| **Self report** |  |  |  |  |  |  |
| Oxford Hip and Knee Questionnaires | Not defined -Patients perception of outcome | Likert (not explicitly stated) | 20 patients interviewed & review of existing questionnaires. 20 items drafted (method of reduction to 20 items not explained in literature) | 20 items tested on 3x20 patients,  reviewed and modified, until final version of 12 items agreed | All 5 response categories  Different wordings | Overall sum |
| WOMAC | Objective of defining the dimensionality of pain and disability. Five dimensions initially. Final version had 3 subscales of pain, stiffness and physical function | Likert    VAS  Numeric rating scale | 100 patients probing 5 dimensions pilot: 41 items | Psychometric, quasi-experimental trials and scaling & statistical analysis - 24 items | Likert: All 5 response categories with same wording  VAS: 0-100mm  NRS: 0-10 | Sum each dimension and overall.  - Other weighting and aggregation methods considered  -Signal method explored but not currently recommended |

Abbreviations: ARA=American Rheumatism Association; CFA=confirmatory factor analysis; EFA=exploratory factor analysis, NRS=numeric rating scale

RA=Rheumatoid arthritis; VAS=Visual analogue scale
